# Understanding the Synthon Preferences in Molecular Ionic Cocrystals of Trimethoprim—An Experimental and Computational Study

**DOI:** 10.1021/acsomega.5c00215

**Published:** 2025-05-05

**Authors:** Lamis Alaa Eldin Refat, Andrea Erxleben

**Affiliations:** † School of Biological and Chemical Sciences, University of Galway, Galway H91TK33, Ireland; ‡ Synthesis and Solid State Pharmaceutical Centre (SSPC), Limerick V94T9PX, Ireland

## Abstract

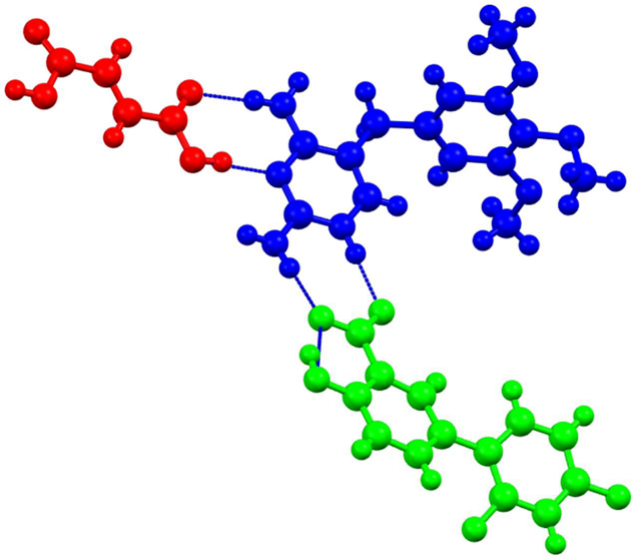

Molecular ionic cocrystals (ICCs) are cocrystals of composition A^–^BH^+^HA or A^–^BH^+^C with charge-assisted hydrogen bonding between A^–^ and BH^+^ and with HA, B, and C being organic solids at ambient temperature. In contrast to the numerous works on the rational design of ternary A·B·C cocrystals, the application of synthon preferences and hierarchies in the synthesis of molecular ICCs is not widely reported. The antibiotic trimethoprim (tmp) readily forms molecular salts with carboxylic acid coformers including nonsteroidal anti-inflammatory drugs. The carboxylate anion interacts with the protonated N1H^+^/C2-NH_2_ site of Htmp^+^ leaving the N3/C4-NH_2_ site as a second binding site for potential ICC formation. In this work, we investigated the synthesis of ternary molecular ICCs of tmp. Solution crystallization experiments led to the single crystal structure of Htmp^+^dif^–^·H_2_fum (dif^–^ = diflunisal anion; H_2_fum = fumaric acid). Hirshfeld surface analysis, molecular electrostatic potential, and site interaction energy calculations were conducted to understand the hydrogen bonding propensity of the N3/C2-NH_2_ site in Htmp^+^X^–^. Proton transfer from HX to the N1 nitrogen of tmp leads to a decrease in the electrostatic potential of N3 and thus to a reduced hydrogen bond acceptor strength. The data obtained in this study highlight the challenges of developing strategies for the rational synthesis of molecular ICCs of complex molecules.

## Introduction

Cocrystallization is now a well-established strategy to manipulate the physicochemical properties of active pharmaceutical ingredients (APIs) without changing their chemical composition.^1^ Examples for marketed pharmaceutical cocrystals are Suglat (Ipragliflozin and l-proline), Steglatoro (Ertugliflozin and *Z*-pyroglutamic acid) and Lexapro (Escitalopram and oxalate).^2^ In particular, the problem of poor aqueous solubility and thus poor bioavailability is being addressed by the design of a suitable cocrystal of the API with a GRAS (generally recognized as safe) coformer. An advanced application of cocrystallization is the development of drug–drug cocrystals in which the GRAS coformer is replaced with a second complementary drug.^3^ The cocrystal of sacubitril and valsartan, sold under the brand name Entresto, was approved by the FDA in 2015 and is one of the world’s top selling drugs.^3^ Dual drug cocrystals offer new opportunities in the formulation of fixed-dose combinations which are supposed to simplify multidrug regimes and improve patient compliance. A number of marketed fixed-dose combinations are included in the World Health Organization’s list of essential medicines, such as levodopa/carbidopa, isoniazid/ethambutol and sulfamethoxazole/trimethoprim.^4^

**Figure 1:**
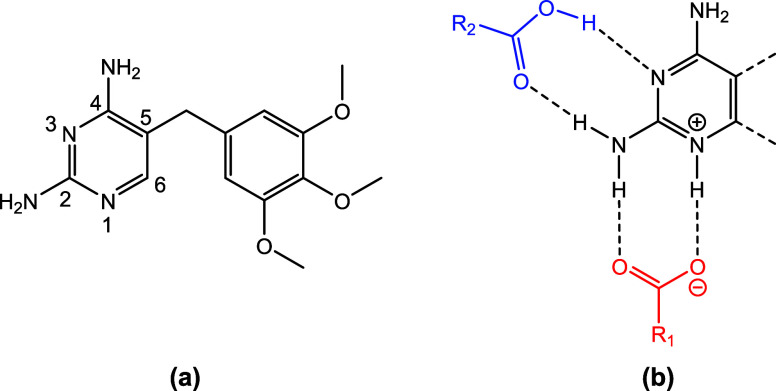
(a) Chemical structure of tmp and (b) design of a nonconjugate acid/base ICC by binding of a second carboxyl coformer to a Htmp^+^ carboxylate.

For the treatment of infections, an antibiotic is often prescribed together with a nonsteroidal anti-inflammatory drug (NSAID) to manage symptoms such as pain, fever and inflammation.^5^ Trimethoprim (tmp, Figure 1a) inhibits bacterial dihydrofolate reductase and is mainly used to treat bladder, kidney and certain ear infections. It readily forms cocrystals or salts with carboxylic acids in which the carboxyl group interacts with the N1 nitrogen and the amino group at the C2 position through a pair of hydrogen bonds.^6−8^ In the case of salt formation proton transfer from the carboxyl group to the N1 nitrogen occurs and the hydrogen bonding is charge-assisted. Many NSAIDs like fenamic acids, diflunisal (Hdif) and diclofenac (Hdic) contain a carboxyl group and are therefore suitable candidates for cocrystallization with tmp. Bhattacharya et al. reported the single crystal structures of the salt hydrates of tmp and mefenamic acid, tolfenamic acid and flufenamic acid and showed that salt formation led to an enhanced solubility of the parent APIs in two of the three salts.^6^

Like many APIs, tmp can interact with coformers through more than one binding site and this can be exploited to obtain higher-order cocrystals. As shown in Figure 1b, the N3,C4-NH_2_ site of the diaminopyrimidine ring in tmp carboxylates presents a potential interaction site for a second carboxyl group. A growing number of ternary cocrystals are described in the literature,^9^ often derived from a ditopic, unsymmetric molecule and built up by using hierarchical intermolecular interactions.^10−15^ These works have provided insight into synthon hierarchies, paving the way to the rational design of multicomponent cocrystals.^16,17^ The attachment of two carboxylic acids that differ in their p*K*_a_ value and thus hydrogen bonding ability to two different binding sites of a molecule was first reported by Aakeroy and co-workers.^18^

In contrast to ternary cocrystals, the rational design of ternary molecular ionic cocrystals of type A^–^BH^+^C is underexplored. Ionic cocrystals (ICCs) are cocrystals of organic molecules and inorganic salts such as NaCl or CaCl_2_^19−24^ whereas molecular ICCs have been defined as cocrystals of organic molecules that contain charge-assisted hydrogen bonds as a result of proton transfer between the coformers and that have an A^–^BH^+^HA or A^–^BH^+^C composition (where C is a solid at ambient temperature).^25^ The terms conjugate acid/base and nonconjugate acid/base ICCs have been proposed for A^–^BH^+^HA and A^–^BH^+^C cocrystals, respectively.^25^ To date, there are very few examples of A^–^BH^+^C molecular ionic cocrystals.^8,25−28^ One of the few works aimed at the targeted synthesis of molecular ICCs was reported by Shunnar et al. in 2020.^25^ They performed computational crystal structure prediction calculations for conjugate and nonconjugate acid/base ICCs derived from 4-dimethylaminopyridine and carboxylic acids.

In this work we investigate the formation of ternary molecular ICCs of tmp with NSAIDs and carboxylic acid coformers. While the design of such a three-component system may look straightforward on paper, the synthesis is in fact a challenging task, as there is a significant risk that the large solubility difference between tmp/NSAID and the carboxylic acid will lead to the precipitation of the individual components or to the competing crystallization of the binary tmp-NSAID cocrystal/salt. Although the cocrystals reported in this work contain APIs, the focus of this study was not on preparing new pharmaceutical materials but on investigating three-component cocrystals of composition A^–^Htmp^+^C. A central question concerned the effect of proton transfer on the synthon preferences of the second binding site of tmp. We therefore performed a screening study for ternary molecular ionic cocrystals containing tmp and two different carboxylic acid coformers. The latter include NSAIDs as well as aliphatic and aromatic di- and tricarboxylic acids. The experimental work was complemented by a theoretical investigation of the hydrogen bonding propensity of the two binding sites of tmp.

## Materials and Methods

### Materials

Trimethoprim (tmp), diclofenac (Hdic), diflunisal (Hdif), oxalic acid (H_2_ox), azeliac acid (H_2_az), pimelic acid (H_2_pim), sebacic aid (H_2_seb), adipic acid (H_2_adi), suberic acid (H_2_sub), aconitic acid (H_3_acn), and 1,2,3-propane tricarboxylic acid (H_3_ptca) were purchased from Tokyo Chemical Industry Europe. Fumaric acid (H_2_fum) was purchased from Fluka Analytical. Maleic acid (H_2_male) was purchased from Merck Millipore. Analytical grade acetonitrile and methanol (Merck Millipore) were used as received.

### Solution Crystallization

0.086 mmol of tmp, 0.086 mmol of coformer 1 and 0.086 mmol of coformer 2 were dissolved in the minimum amount of solvent (acetonitrile or methanol). The solutions were allowed to slowly evaporate at room temperature. Details on the cocrystallization experiments can be found in Table S1.

### Single Crystal X-ray Analysis

Single crystal X-ray analyses of (Htmp^+^)(dif^–^)·H_2_fum, Htmp^+^dif^–^·H_2_O, Htmp^+^dic^–^·1.5H_2_O, (Htmp^+^)(dif^–^)·CH_3_OH^.^0.5H_2_O, Htmp^+^Hseb^–^, Htmp^+^Hmale^–^·CH_3_CN, (Htmp^+^)(sub^2–^)_0.5_(H_2_sub)_0.5_·H_2_O, (Htmp^+^)(adi^2–^)_0.5_(H_2_adi)_0.5_·2H_2_O, Htmp^+^H_2_ptca^–^·CH_3_CN, and (Htmp^+^)_2_Hacn^2–^·3H_2_O were performed with an Oxford Diffraction Xcalibur system (Oxfordshire, U.K.). The crystal structures were solved by direct methods and refined using SHELXT^29^ and SHELXL 2018/3^30^ within the Oscail^31^ and Olex2^32^ program suites. The N–H and C–OH hydrogens were located in the difference Fourier maps and refined isotropically. The C–H hydrogen atoms were placed in geometrically idealized positions and refined using a riding model. All structures except for Htmp^+^Hmale^–^·CH_3_CN and (Htmp^+^)_2_Hacn^2–^·3H_2_O contained some form of disorder. The disordered atoms were modeled over multiple positions with fixed occupancies. Drawings were produced with Mercury^33^ and with ORTEX and POGL embedded in Oscail.^31^ Crystallographic data and details of refinement are listed in Table 1.

**Table 1:** Crystal Data of Htmp^+^dif^–^·H_2_fum, Htmp^+^dif^–^·H_2_O, (Htmp^+^)(dif^–^)·CH_3_OH.0.5H_2_O, Htmp^+^dic^–^·1.5H_2_O, Htmp^+^male^–^·CH_3_CN, Htmp^+^(sub^2–^)_0.5_(H_2_sub)_0.5_·H_2_O, Htmp^+^(adi^2–^)_0.5_(H_2_adi)_0.5_·2H_2_O, Htmp^+^H_2_ptca^–^·CH_3_CN, (Htmp^+^)_2_Hacn^2–^·3H_2_O, and Htmp^+^Hseb^–^

	Htmp^+^dif^–^·H_2_fum	Htmp^+^dif^–^·H_2_O	(Htmp^+^)(dif^–^)· CH_3_OH.0.5H_2_O	Htmp^+^dic^–^·1.5 H_2_O	Htmp^+^Hmale^–^·CH_3_CN	Htmp^+^(sub^2–^)_0.5_ (H_2_sub)_0.5_·H_2_O	Htmp^+^(adi^2–^)_0.5_ (H_2_adi)_0.5_·2H_2_O	Htmp^+^H_2_ptca^–^·CH_3_CN	(Htmp^+^)_2_Hacn^2–^·3H_2_O	Htmp^+^Hseb^–^
formula	C_31_H_30_F_2_N_4_O_10_	C_27_H_28_F_2_N_4_O_7_	C_56_H_62_F_4_N_8_O_15_	C_56_H_64_Cl_4_N_10_O_13_	C_20_H_25_N_5_O_7_	C_22_H_34_N_4_O_8_	C_20_H_32_N_4_O_9_	C_22_H_29_N_5_O_9_	C_34_H_48_N_8_O_15_	C_24_H_36_N_4_O_7_
*M*_r_	656.59	558.53	1163.13	1226.97	447.45	482.53	472.49	507.50	808.80	492.57
crystal color and habit	colorless block	colorless block	colorless block	colorless block	colorless block	colorless block	colorless block	colorless block	colorless block	colorless block
crystal system	monoclinic	triclinic	triclinic	triclinic	monoclinic	triclinic	triclinic	triclinic	triclinic	orthorhombic
space group	*P*2_**1**_/*c*	*P*-1	*P*-1	*P*-1	*P*2_**1**_/*n*	*P*-1	*P*-1	*P*-1	*P*-1	*Pbca*
*a* [Å]	15.0134(5)	10.6353(8)	10.8200(4)	12.1098(6)	5.5006(4)	5.6318(3)	5.3417(3)	5.5657(4)	12.2024(6)	13.3747(10)
*b* [Å]	13.9579(4)	11.2679(9)	14.1385(5)	15.2769(8)	28.687(2)	14.2111(8)	14.7175(10)	12.5168(15)	12.7126(6)	17.5821(17)
*c* [Å]	14.4899(6)	12.7457(11)	18.9120(8)	17.7221(9)	14.1515(11)	16.2960(10)	15.8321(10)	18.226(2)	13.9100(6)	22.6176(13)
α [°]	90	94.896(7)	78.956(3)	84.237(4)	90	98.047(5)	107.439(6)	85.327(10)	73.348(4)	90
β [°]	95.861	113.262(8)	83.236(3)	77.701(4)	100.684(8)	91.483(4)	94.214(5)	85.089(8)	87.872(4)	90
γ [°]	90	102.275(7)	74.624(3)	69.621(5)	90	94.122(4)	93.652(5)	86.558(8)	76.478(4)	90
*V* [Å^3^]	3020.57(18)	1346.6(2)	2731.05(19)	3001.6(3)	2194.3(3)	1287.20(13)	1179.43(13)	1259.0(2)	2008.95(17)	5318.6(7)
*Z*	4	2	2	2	4	2	2	2	2	8
*D*_calc_ (g cm^–3^)	1.444	1.378	1.414	1.358	1.354	1.245	1.330	1.339	1.337	1.230
temperature (K)	298.5(9)	293.0(2)	149.9(1)	293.0(2)	149.9(2)	293.0(2)	293.0(2)	293.0(2)	293.0(2)	293.0(2)
no. measd. reflections	26336	9997	24423	26124	18987	9436	10140	9385	17393	38879
no. unique refl. (*R*_int_)	7271 (0.0306)	4933 (0.0474)	12681(0.0262)	13770 (0.0222)	5263 (0.0709)	4702 (0.0314)	5437 (0.0251)	4612 (0.0568)	9325 (0.0279)	4676 (0.065)
no. obs. reflections	4847	2829	8683	8513	2886	3289	4114	2760	5980	2672
final *R*_1_, *w*R**_2_ (obs. refl.)	0.0514, 0.1116	0.0542, 0.1117	0.0544, 0.1322	0.0591, 0.1540	0.0734, 0.1616	0.0544, 0.1399	0.0548, 0.1397	0.0935, 0.2527	0.0571, 0.1396	0.0672, 0.1628
goodness-of-fit (obs. refl.)	1.018	1.012	1.027	1.047	1.044	1.024	1.040	1.049	0.988	1.015

### Thermal Analysis

Differential scanning calorimetry (DSC) and thermogravimetric analysis plots were recorded in open aluminum crucibles with an STA625 thermal analyzer (RheometricScientific, Piscataway, New Jersey). Samples were heated under N_2_ purge at 10 °C/min. An indium standard was used for calibration.

### Theoretical Calculations

#### Hirshfeld Surface Analysis

The Hirshfeld surfaces and 2D fingerprint plots were generated from the CIFs of the cocrystals with Crystal Explorer 17.5.^34^ The Hirshfeld surfaces were mapped on *d*_norm_. The intermolecular interactions were quantified by filtering the 2D fingerprint plots (*d*_i_ vs *d*_e_) by element.

#### Molecular Electrostatic Potential (MEP) Analysis and Site Interaction Energies

The geometry optimization of the structures and the calculation of the electron densities was performed using density functional theory (DFT) with the ORCA quantum chemistry package.^35^ Calculations were conducted at the wB97X-D3/6–31G** level which combines a hybrid functional with empirical dispersion correction to accurately model noncovalent interactions. The Conductor-like Polarizable Continuum Model (CPCM) was employed to account for solvent effects during the optimization process. The wave function files were obtained from the ORCA GBW files and used to calculate the MEP maps of the optimized structures with Multiwfn.^36,37^ The MEP was mapped onto the 0.001 au electron density isosurface and plots were produced with Visual Molecular Dynamics (VMD 1.9.3) software.^38^ The hydrogen bond donor (α) and acceptor (β) parameters were calculated using the equations
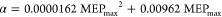
1
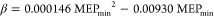
2where MEP_max_ and MEP_min_ are the MEP maxima and minima in kJ mol^–1^, respectively.^39^

The interaction site pairing energy between a hydrogen bond donor and acceptor is given by
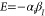
3and the total pairing energy of the crystal is given by^39^
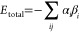
4

## Results and Discussion

### Screening for Ternary ICCs

**Figure 2:**
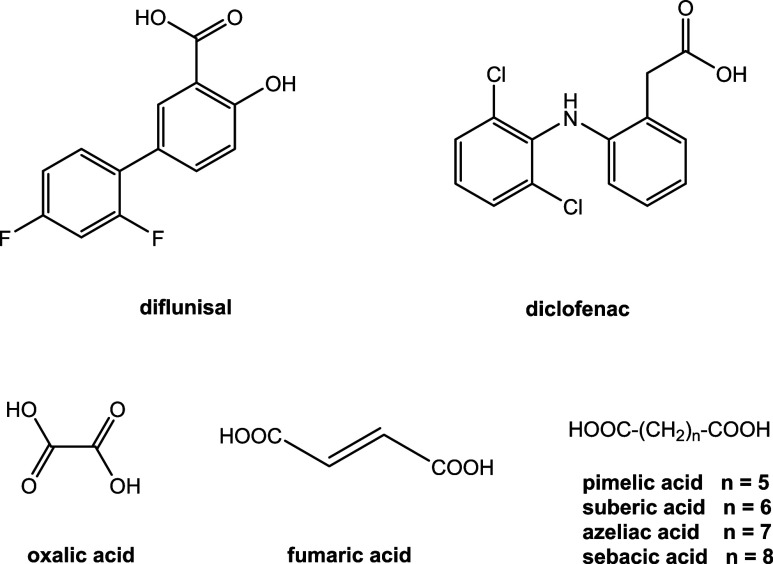
Chemical structures of the NSAIDs and carboxylic acid coformers used in this study.

We first performed a screening study for ternary ICC formation. The NSAIDs used were Hdif and Hdic and fumaric acid (H_2_fum), oxalic acid (H_2_ox), azeliac acid (H_2_az), pimelic acid (H_2_pim), suberic acid (H_2_sub), and sebacic aid (H_2_seb) were selected as the second coformer (Figure 2). Solution cocrystallization experiments were performed on a 50 mg scale by slow evaporation of solutions containing tmp and the respective NSAID and carboxylic acid coformer in a 1:1:1 molar ratio. In total, 12 different combinations were screened using two different solvents (Table S1). This resulted in one new ternary cocrystal; (Htmp^+^)(dif^–^)^.^H_2_fum (**1**). Unsuccessful combinations gave powders or crystals unsuitable for structure determination, crystals of individual components or binary salts (Table S1).

**Figure 3:**
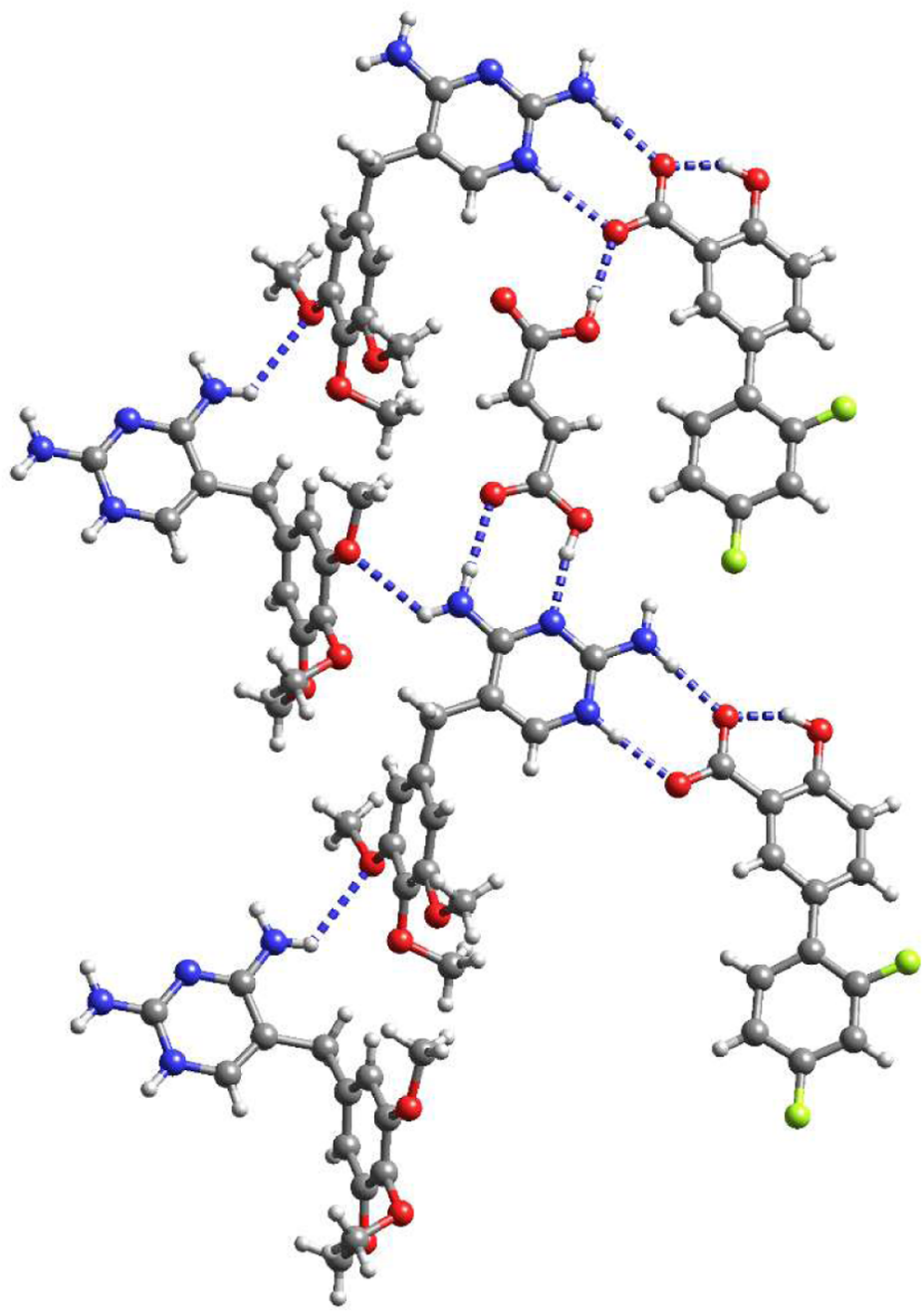
X-ray structure and H bonding motif of (Htmp^+^)(dif^–^)·H_2_fum (**1**).

The X-ray structure of **1** is shown in Figure 3. The asymmetric unit contains a Htmp^+^ cation, a dif^–^ anion and a neutral H_2_fum molecule. Dif^–^ forms the typical *R*_2_^2^(8) heterosynthon through pairwise hydrogen bonding with the amino group at C2 and the protonated N1 nitrogen of the pyrimidine ring. Evidence for the proton transfer from the carboxyl group to the pyrimidine ring are the C–O bond lengths that are equal within the standard deviations [1.255(2) and 1.264(2) Å]. One of the carboxyl groups of H_2_fum interacts with the N3 nitrogen and the amino group at C4 [*R*_2_^2^(8)]. The second carboxyl group of H_2_fum donates a single hydrogen bond to one of the carboxylate oxygens of the dif^–^ anion. One of the methoxy groups of Htmp^+^ accepts a hydrogen bond from the C4-amino group of a neighboring Htmp^+^ cation.

The DSC plot of **1** shows a single endotherm at 180.3 °C due to melting of the cocrystal followed by decomposition at 270 °C (Figure S1). It was not possible to isolate bulk quantities of the ternary cocrystal. When the scale of the solution crystallization experiment was increased from 50 to 200 mg, the X-ray powder pattern of the isolated material had broad peaks and was inconclusive. The DSC plot showed multiple thermal events suggesting a mixture. To better understand the cocrystal formation we performed a detailed study of the properties of the two binding sites in tmp.

### Molecular Electrostatic Potential (MEP) Surface Analysis

The MEP gives insight into the nucleophilicity of interaction sites and has been used by Hunter to rank the relative hydrogen bond donor and acceptor strengths.^39,40^ The more positive the MEP value, the better is the hydrogen bond donor ability while a more negative value corresponds to a better hydrogen bond acceptor. Etter’s rules state that the best hydrogen bond donor interacts with the best hydrogen bond acceptor, the second best hydrogen bond donor interacts with the second best acceptor and so on until all possible hydrogen bonds are formed.^41^

**Figure 4:**
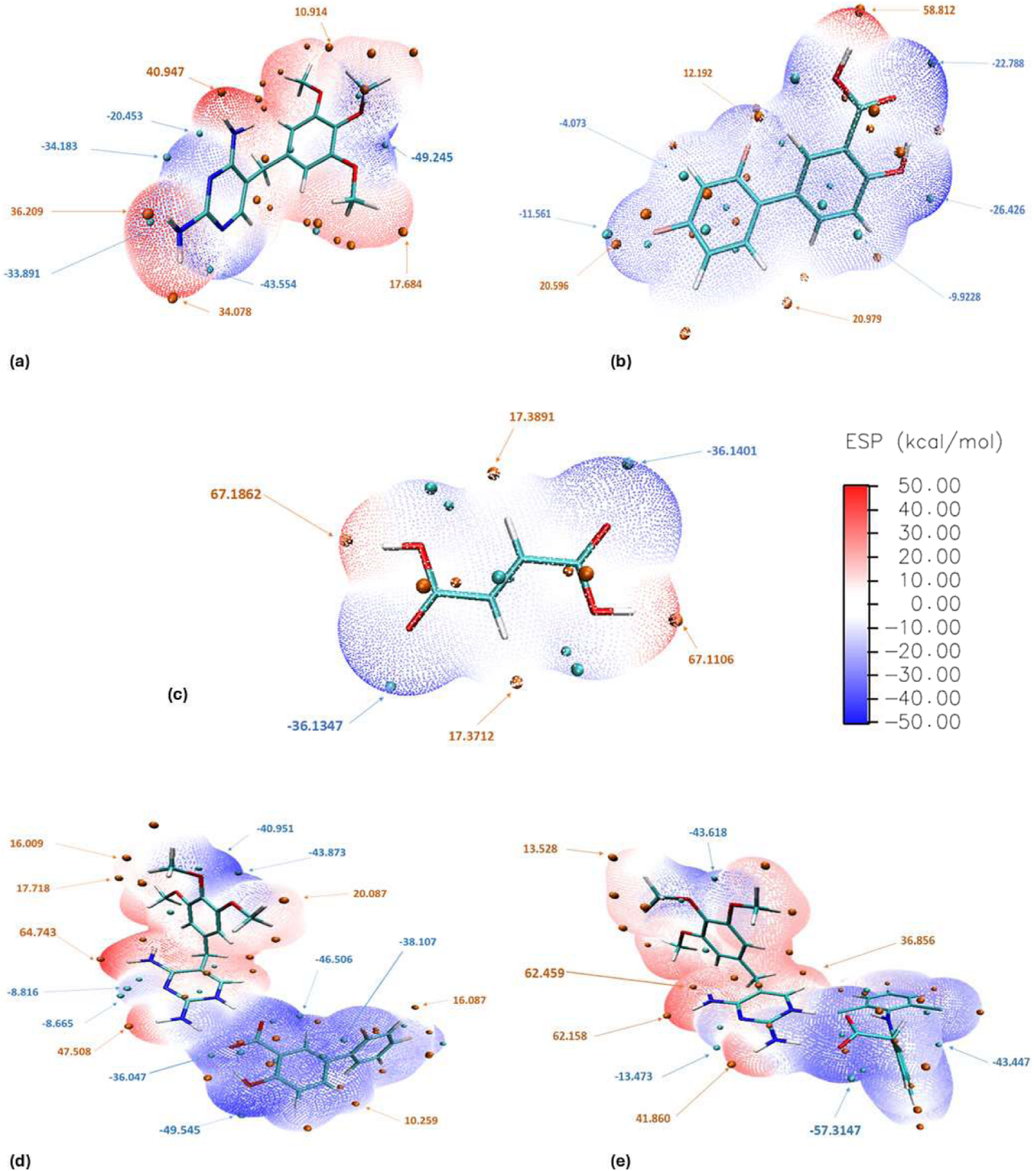
MEPs (kcal mol^–1^) of (a) tmp, (b) Hdif, (c) H_2_fum, (d) Htmp^+^dif^–^, and (e) Htmp^+^dic^–^ mapped onto the 0.001 au electron density surface. Maxima and minima of the MEP are shown as red and cyan spheres.

The MEP surface of tmp calculated at the wB97X-D3/6–31G** level of theory is shown in Figure 4a. The N1 nitrogen has the most negative electrostatic potential (−43.55 kcal mol^–1^). The electrostatic potential of N3 is 9.4 kcal mol^–1^ less negative than that of N1. These values are in line with the MEP surface previously calculated at the B3LYP-D3/6–311G(d,p) level by Hu et al.^42^ The hydrogen atoms of the carboxyl groups of H_2_fum have a more positive electrostatic potential than the carboxyl hydrogen of Hdif (67.15 vs 58.81 kcal mol^–1^, Figure 4b,c), while the electrostatic potential at the carbonyl oxygens is more negative in H_2_fum compared to Hdif (−36.14 vs −22.79 kcal mol^–1^). The less negative electrostatic potential at the carbonyl site in Hdif results from the intramolecular hydrogen bond between the phenol group and C=O. Based on the MEP calculations and Etter’s rule one would expect H_2_fum to interact with the N1,C2-NH_2_ site and Hdif to bind to the second binding site in contrast to the observed structure. As the interaction involves proton transfer, the p*K*_a_ values of the coformers are relevant. The p*K*_a1_ value of H_2_fum is 3.03,^43^ while values of 2.69^44^ and 3.30^45^ can be found in the literature for Hdif. The lower the p*K*_a_ value, the greater is the ability of proton transfer. Thus, no clear correlations between the p*K*_a_ values, calculated electrostatic potentials of the carboxyl hydrogens, the binding site preferences and the protonation states of the two coformers in the ternary cocrystal can be established.

We then computed the MEP for the Htmp^+^X^–^ entities in the binary salts Htmp^+^dif^–^·H_2_O and Htmp^+^dic^–^·1.5H_2_O that were also prepared and structurally characterized in this work (Supporting Information). As shown in Figure 4d,e, on salt formation the electrostatic potential at the *N3* site decreases (becomes less negative) by about 20 kcal mol^–1^ and the electrostatic potential around the hydrogens of the exocyclic amino group at C4 increases (becomes more positive) by about the same value. That is, the hydrogen bond donor strength of the amino group is enhanced compared to neutral tmp while N3 becomes a weaker hydrogen bond acceptor. Azeliac acid forms a cocrystal with tmp in which the COOH···N1,C2-NH_2_*R*_2_^2^(8) motif does not involve proton transfer.^8^ Calculation of the MEP surface (Figure S2) indicates that in this case of neutral adduct formation the electrostatic potential at the N3 and C4-NH_2_ site changes marginally only (<5 kcal mol^–1^) compared to tmp.

### Site Interaction Energy

Hunter suggested that the electrostatic interaction is the dominant contribution to the enthalpy of intermolecular interactions, while repulsion, induction and dispersion contributions are negligible.^39,40^ He used the α- and β-parameters to quantify and extract the strength of a hydrogen bonding site from the MEP map. According to Hunter the interaction site pairing energy between a hydrogen bond donor and acceptor can be estimated using eq 3 in the Materials and Method Section.^39^

To calculate the interaction site pairing energy for the COOH···N3,C4-NH_2_ interaction in the ternary cocrystal **1**, the α,β-parameters of the C=O, C–OH, N3 and C4-NH_2_ sites of the coformer and of the Htmp^+^dif^–^ entity were calculated as described in the experimental section. Using eq 3 and the α- and β-parameters listed in Table 2, a pairing energy of −4.8 kcal mol^–1^ was obtained for the interaction of fumaric acid with the N3,C4-NH_2_ site of Htmp^+^dif^–^. This is higher than the pairing energy of −0.9 kcal mol^–1^ computed for the C4-NH_2_···N3 homodimer interaction in Htmp^+^dif^–^. While this supports the conclusion that the COOH···N3,C4-NH_2_ synthon is preferred over the C4-NH_2_···N3 homosynthon, the application of the interaction site pairing energy model to predict synthon hierarchies may be an oversimplified approach in the case of tmp, as the homodimer can be stabilized through a bridging water molecule that hydrogen bonds to the amino groups at C2 and C4. In fact, this type of hydrogen bonding is observed in the crystal structure of Htmp^+^dif^–^^.^H_2_O (Supporting Information).

**Table 2:** α- and β-Parameters for the Interaction Sites in tmp and the Coformers

α				β		
	carboxyl hydrogen	C4-NH_2_	C2-NH_2_	C=O (COO^–^)	C=O (COOH)	N3
H_2_fum	4.0			4.7		
Htmp^+^dif^–^		3.8	2.6			0.5
Htmp^+^dic^–^		3.6	2.2			1.0
Htmp^+^H_2_tma^–^	3.2	3.7	2.4		8.3	0.7
Htmp^+^Hadi^–^	2.9	3.8	2.2	14.6	9.6	1.0

### Interactions of the N3,C4-NH_2_ Binding Site in Binary tmp Carboxylates

To gain more insight into the binding preferences of the second binding site of tmp in Htmp^+^ carboxylates, we thought it instructive to survey binary carboxylates that contain a neutral COOH group in addition to the COO^–^ group forming the *R*_2_^2^(8)motif with N1H^+^/C2-NH_2_. Specifically, we wanted to see if the N3,C4-NH_2_ site of a neighboring Htmp^+^ interacts with this neutral carboxyl group. The cocrystals/salts included in this survey comprise structures from the CSD and new structures prepared in our lab which contain a neutral dicarboxylic acid, a dicarboxylic acid monoanion or a tricarboxylic acid dianion as well as ionic cocrystals of composition Htmp^+^X^–^^.^HX or (Htmp^+^)_2_X^2–^^.^H_2_X (Table 3).

**Table 3:** Binary 1:1 Cocrystals/salts of tmp Containing a Neutral Dicarboxylic Acid, a Dicarboxylic Acid Monoanion or a Tricarboxylic Acid Dianion, and Ionic Cocrystals of Composition Htmp^+^X^–^·HX or (Htmp^+^)_2_X^2–^·H_2_X

composition^a^	cocrystal/salt/ionic cocrystal	N1,C2-NH_2_ synthon	N3,C4-NH_2_ synthon	ref
Htmp^+^bz^–^·Hbz form I	ionic cocrystal	*R*_1_^2^(6)	C4-NH_2_···N3 homosynthon	CUCSEY01
Htmp^+^bz^–^·Hbz form II	ionic cocrystal	*R*_2_^2^(8)	C4-NH_2_···N3 homosynthon	CUCSEY10
Htmp^+^Hadi^–^	salt	*R*_2_^2^(8)	synthon III	SEMNEE
(Htmp^+^)_2_ter^2–^·H_2_ter	ionic cocrystal	*R*_2_^2^(8)	C4-NH_2_···N3 homosynthon	VADVOM
Htmp^+^Hglu^–^	salt	*R*_2_^2^(8)	synthon III	CACBOY
Htmp^+^Hfum^–^	salt	N1H^+^...^–^OOC	synthon III	CURSAL
Htmp^+^Hketo^–^·0.5H_2_O	salt	*R*_1_^2^(6)	C4-NH_2_···N3 homosynthon	KAXMIJ
Htmp^+^Hmale^–^·CH_3_CN	salt	*R*_2_^2^(8)	C4-NH_2_···N3 homosynthon	this work
			C2-NH_2_···O = (OH)C	
			C4-NH_2_···O = (OH)C	
Htmp^+^Hmale^–^	salt	*R*_2_^2^(8)	C4-NH_2_···N3 homosynthon	QIKDIX
			C2-NH_2_···O = (OH)C	
			C4-NH_2_···O = (OH)C	
(Htmp^+^)(sub^2–^)_0.5_ (H_2_sub)_0.5_·H_2_O	ionic cocrystal	*R*_2_^2^(8)	C4-NH_2_···N3 homosynthon	this work
(Htmp^+^)(adi^2–^)_0.5_ (H_2_adi)_0.5_·2H_2_O	ionic cocrystal	*R*_2_^2^(8)	C4-NH_2_···N3 homosynthon	this work
Htmp^+^H_2_ptca^–^·CH_3_CN	salt	*R*_2_^2^(8)	C4-NH_2_···O = (OH)C, C2-NH_2_···O = (OH)C & C4-NH_2_···N3 homosynthon	this work
(Htmp^+^)_2_(Hacn^2–^)·3H_2_O	salt	*R*_2_^2^(8)	molecule A: N3···H_2_O & C4-NH_2_...COOH	this work
			molecule B:	
			C2-NH_2_···–OOC	
			C4-NH_2_···H_2_O	
Htmp^+^Hmalo^–^	salt	*R*_2_^2^(8)	C4-NH_2_···OCH_3_	this work
Htmp^+^Hseb^–^	salt	*R*_2_^2^(8)	C4-NH_2_···OCH_3_	this work
			C2-NH_2_···OCH_3_	
			C4-NH_2_···–OOC	
Htmp^+^H_2_tma^–^·3H_2_O	salt	*R*_2_^2^(8)	C4-NH_2_,N3···HOOC	KUMJIP
Htmp^+^Haz^–^	salt	*R*_2_^2^(8)	C4-NH_2_···OCH_3_	KAXNAC
			C4-NH_2_···–OOC	
tmp.H_2_az	cocrystal	*R*_2_^2^(8)	C4-NH_2_,N3···HOOC	KAXMOP
tmp.H_2_pim·0.5CH_3_CN	cocrystal	*R*_2_^2^(8)	C4-NH_2_,N3···HOOC	KAXNOQ

a Hbz = benzoic acid; H_2_adi = adipic acid; H_2_ter = terephthalic acid; H_2_glu = glutaric acid; H_2_fum = fumaric acid; H_2_keto = α-ketoglutaric acid; H_2_malo = malonic acid; H_2_az = azelaic acid; H_2_pim = pimelic acid; H_2_male = maleic acid; H_3_acn = aconitic acid; H_3_tma = trimesic acid

**Figure 5:**
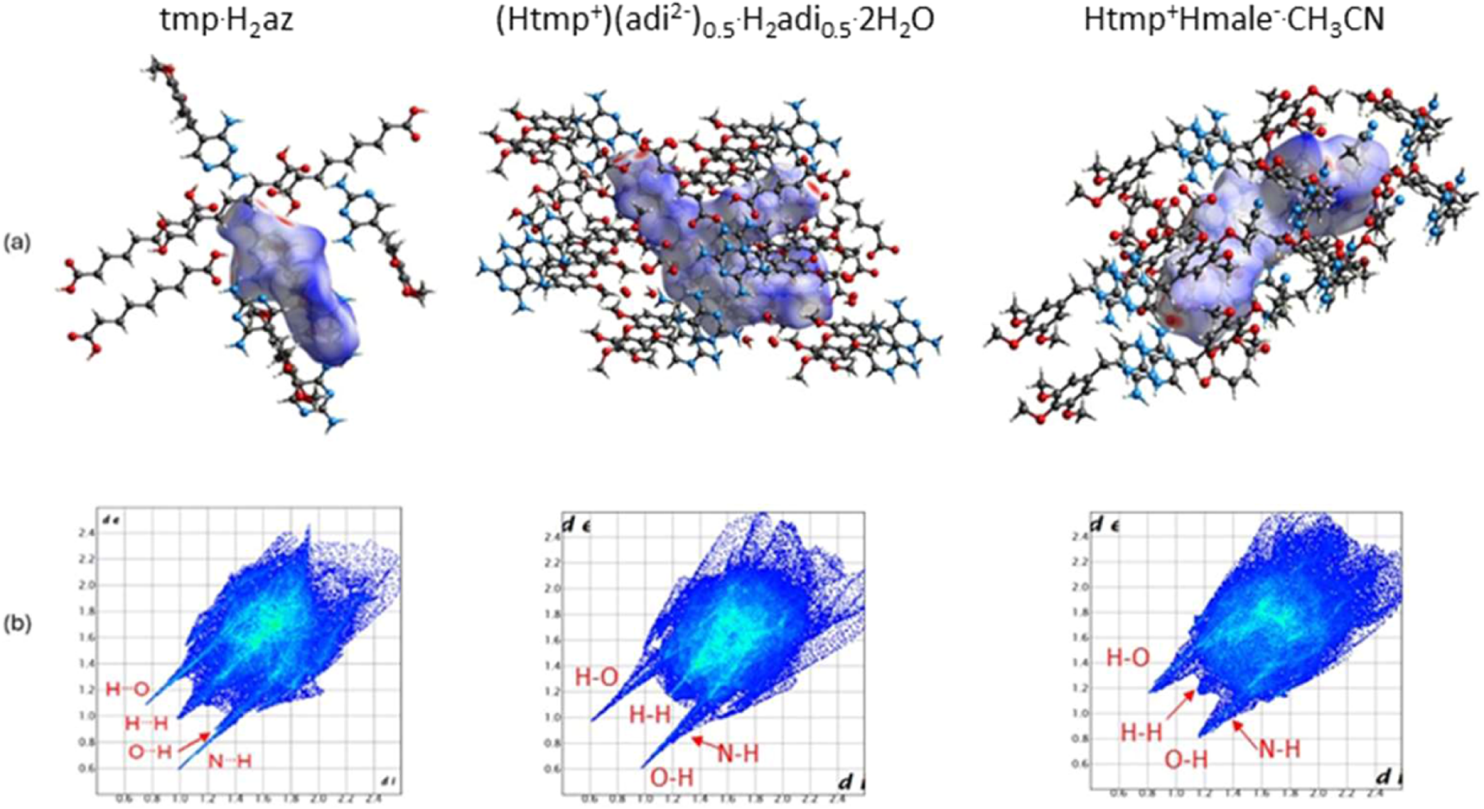
(a) Hirshfeld surface (*d*_norm_) of tmp in tmp.H_2_az (KAXMOP^8^), (Htmp^+^)(adi^2–^)_0.5_(H_2_adi)_0.5_·2H_2_O and Htmp^+^Hmale^–^·CH_3_CN and molecules with atoms within a distance of 3.5 Å outside the Hirshfeld surface map. The red spots represent H bonding, white regions represent weak interactions (H···H, C···H) and blue regions represent no important contacts. (b) 2D fingerprint plots (*d*_i_ vs *d*_e_, where *d*_i_ and *d*_e_ are the distances to the nearest atom inside and outside the surface, respectively).

First, we performed Hirshfeld surface analysis to gain a general overview of the intermolecular interactions. Representative examples of the Hirshfeld surfaces and 2D fingerprint plots of the binary carboxylates are shown in Figure 5. Hirshfeld surface analysis allows the quantification of the intermolecular interactions by filtering the 2D fingerprint plots by specific atom pairs.^46^ In all salts H···H contacts make up the largest percentage of the 2D fingerprint plot (39.9–60.2%). O···H/H···O and N···H/H···N hydrogen bonds appear as sharp spikes and account for 19.7–37.7 and 3.8–12.6% of the interactions, respectively (Table S2).

**Figure 6:**
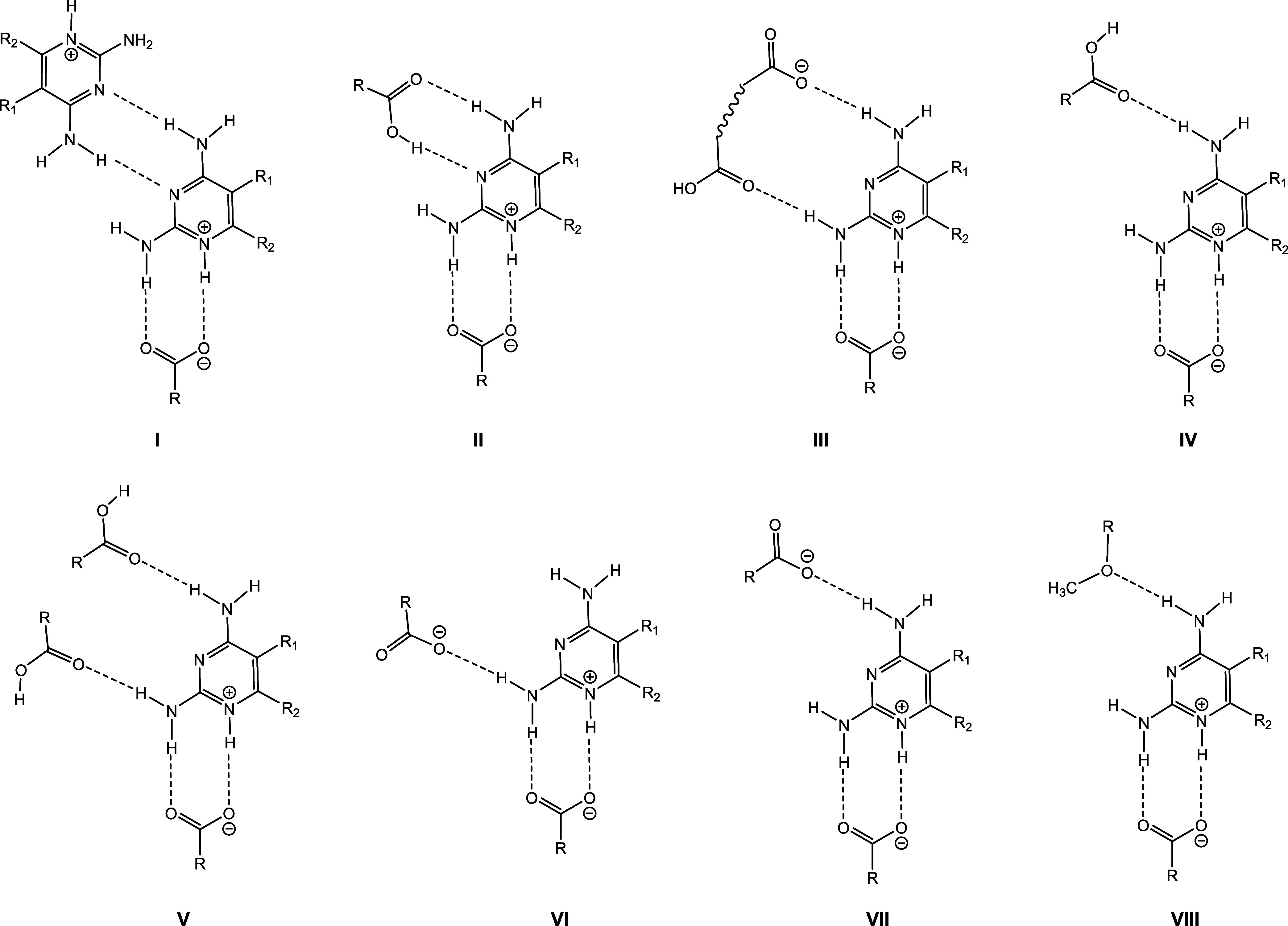
Binding motifs observed in binary 1:1 cocrystals/salts of tmp containing a neutral dicarboxylic acid, a dicarboxylic acid monoanion or a tricarboxylic acid dianion and ionic cocrystals of composition (tmp^+^)_2_X^2–^^.^H_2_X.

Next, we analyzed the main synthons in the single crystal structures. Except for (Htmp^+^)(Hketo^–^)·0.5H_2_O (H_2_keto = α-ketoglutaric acid), Htmp^+^bz^–^·0.5Hbz form I (Hbz = benzoic acid), and Htmp^+^Hfum^–^ all structures feature the N1H^+^,C2-NH_2_···^–^OOC *R*_2_^2^(8) motif as the primary synthon (or the N1,C2-NH_2_···HOOC *R*_2_^2^(8) motif in the case of tmp^.^H_2_az and tmp^.^H_2_pim^.^0.5CH_3_CN). The synthon between Hketo^–^ and bz^–^ and the N1,C2-NH_2_ site of tmp is a *R*_1_^2^(6)motif, while a single N1H^+^···^–^OOC hydrogen bond is present in Htmp^+^Hfum^–^. The binding motifs observed for the N3,C4-NH_2_ site in the binary compounds are shown in Figure 6. While in both cocrystals that contain a neutral tmp (tmp^.^H_2_az and tmp^.^H_2_pim^.^0.5CH_3_CN) the N3,C4-NH_2_ site interacts with the second COOH group of the dicarboxylic acid, the COOH···N3,C4-NH_2_ interaction is only observed in one of the 14 salt/ionic cocrystal structures. Seven of the salts feature the N3,C4-NH_2_ homodimer. In six cases, the N3 nitrogen does not participate in hydrogen bonding. In these structures NH_2_···O=C single hydrogen bonds or a ‘clamp-like’ motif involving both the amino group at C2 and at C4 are present (synthon III, Figure 6).

To rationalize the synthon preferences of the C2-NH_2_,N3,C4-NH_2_ binding site we calculated the MEP, the α,β-parameters (Table 2) and the pairing energies for two representative examples; Htmp^+^H_2_tma^–^^.^3H_2_O (KUMJIP) and Htmp^+^Hadi^–^ (SEMNEE) that have synthon II and III, respectively. The pairing energy for synthon III in SEMNEE is 16.4 kcal mol^–1^ which is 7.9 kJ mol^–1^ higher than the pairing energy for synthon II in Htmp^+^H_2_tma^–^·3H_2_O.

**Figure 7:**
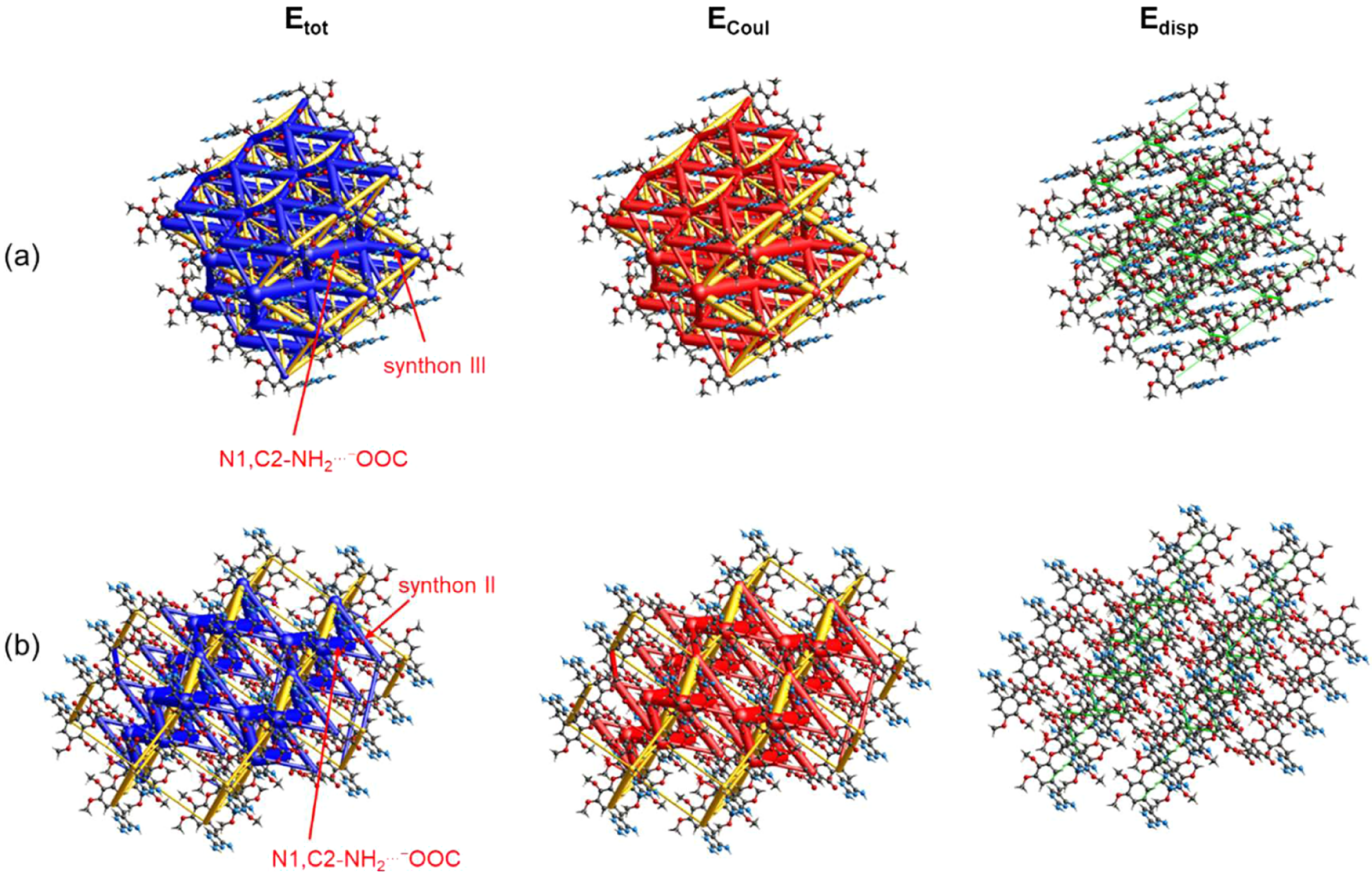
Energy framework diagrams for the total (*E*_tot_), Coulomb (*E*_coul_), and dispersion (*E*_disp_) energies for (a) Htmp^+^Hglu^–^ (CACBOY) and (b) Htmp^+^H_2_tma^–^·3H_2_O (KUMJIP). The cylinder scale is 25 and the cut-offs are 50 for *E*_tot_ and *E*_coul_ and 20 for *E*_disp_. The energies were calculated at the HF/3–21G level of theory.

We further studied the strength of the *R*_2_^2^(8) and “clamp” interaction using Crystal Explorer *17.5*.^34^Figures 7 and S6 show the energy frameworks for Htmp^+^H_2_tma^–^·3H_2_O (KUMJIP), Htmp^+^Hadi^–^ (SEMNEE), and Htmp^+^Hglu^–^ (CACBOY). As expected, the strongest interaction in all three structures is the primary N1,C2-NH_2_···^–^OOC synthon. Synthon III represents the second strongest interaction in Htmp^+^Hglu^–^ and Htmp^+^Hadi^–^. A comparison of the energy framework diagrams confirms that the N3,C4-NH_2_···^–^OOC *R*_2_^2^(8) interaction (synthon II) in Htmp^+^H_2_tma^–^·3H_2_O is considerably weaker than the aforementioned synthon III interaction, consistent with the low nucleophilicity of the *N3* site in the Htmp^+^X^–^ entity. The pairwise total interaction energies (*E*_tot_) and their partitioning into electrostatic (*E*_coul_), polarization (*E*_pol_), dispersion (*E*_disp_) and repulsion (*E*_rep_) contributions are provided in Tables S3–S5. While there is a clear discrepancy between the *E*_coul_ values and the site interaction energies derived from the MEPs and discussed above, both approaches indicate that synthon III is favored over synthon II.

Taken together, all calculations support the observed reluctance of the C2-NH_2_,N3,C4-NH_2_ binding site in Htmp^+^X^–^ dicarboxylates to form hydrogen bonding patterns that involve COOH···N3 interactions. If X^–^ is a monodeprotonated carboxylic acid with a suitable (CH_2_)*_n_*-chain length, synthon III that “leaves out” N3 seems to be a very favorable interaction. In the case of Htmp^+^Hglu^–^ and Htmp^+^Hadi^–^ (*n* = 3, 4) *E*_tot_ was calculated as −79.5 and −81.6 kcal mol^–1^, respectively.

## Conclusions

In this work we have shown that two different carboxylic acid coformers can be attached to the two distinct *N*_amino_,*N*_pyrimidine_ interaction sites in tmp. As one of the *R*_2_^2^(8) motifs formed involves proton transfer, the resulting ternary cocrystal Htmp^+^dif^–^·H_2_fum (**1**) can be classified as a nonconjugate acid/base molecular ionic cocrystal. Although interaction site pairing energy calculations suggest that the COOH···N3,C4-NH_2_*R*_2_^2^(8) motif is stronger than the N3···C4-NH_2_*R*_2_^2^(8) motif in the binary parent salt, **1** was the only ternary ICC obtained in a screening study with 12 coformer combinations. This is arguably a low yield. MEP calculations indicate that proton transfer from the coformer at the N1,C2-NH_2_ binding site reduces the hydrogen bond accepting ability of N3. It is noteworthy that except for oxalic acid all other acids used in the screening experiments are weaker acids, thus weaker hydrogen bond donors than H_2_fum which may explain why the binary salts (Htmp^+^)(dif^–^)·CH_3_OH·0.5H_2_O and (Htmp^+^)_2_(dif^–^)_2_·H_2_O were obtained in these cases instead of a ternary ICC (Table S1). From ternary mixtures of oxalic acid, Hdif and tmp, very thin fibers crystallized that were unsuitable for single crystal X-ray analysis. The analysis of binary salts showed that the formation of two C-NH_2_···O=C hydrogen bonds should be a more favorable interaction for the C2-NH_2_,N3,C4-NH_2_ site than the COOH···N3,C4-NH_2_*R*_2_^2^(8) motif. While we attempted to rationalize the interactions of the C2-NH_2_,N3,C4-NH_2_ binding site of the Htmp^+^dif^–^ entity using MEP and site interaction energy calculations, the role of packing and the importance of the sum of all interactions in the structure (which include interactions with solvent molecules of crystallization) have to be kept in mind. Furthermore, the relative solubilities of the individual components, possible binary combinations and the ternary system must not be overlooked as a determining factor for the successful synthesis of a three-component system. In summary our study emphasizes the challenges of rationally designing ternary ICCs of complex molecules with multiple hydrogen bonding and synthon possibilities.

## Supporting Information Available

Details of the solution crystallization experiments; additional figures and descriptions of X-ray structures; DSC plot of **1**; figures of MEP surfaces (PDF)
