# Supplementary material for: Understanding the Synthon Preferences in Molecular Ionic Cocrystals of Trimethoprim—An Experimental and Computational Study
Source: ACS Omega. 2025 May 5;10(18):18709–19. doi: 10.1021/acsomega.5c00215 (PMC12079235; doi:10.1021/acsomega.5c00215)
Supplement: Supplementary file 1 — ao5c00215_si_011.pdf [file ao5c00215_si_011.pdf]

Understanding the Synthon Preferences in Molecular Ionic Cocrystals of Trimethoprim – An Experimental and Computational Study

Lamis Alaa Eldin Refat[1,2] and Andrea Erxleben[1,2,*]

[1] School of Biological and Chemical Sciences, University of Galway, Galway, H91TK33, Ireland

[2] Synthesis and Solid State Pharmaceutical Centre (SSPC), Limerick, V94T9PX, Ireland

*Corresponding author email address: andrea.erxleben@nuigalway.ie (AE)

Supporting Information

Table S1. Solution cocrystallization experiments of tmp. In all experiments, equimolar ratios were used.

Coformer 1	Coformer 2	Solvent	Result
Diflunisal	Oxalic acid	CH_3OH	Thin fibers
Diflunisal	Oxalic acid	CH_3CN	Thin fibers
Diflunisal	Fumaric acid	CH_3OH	$(Htmp^+)(dif^-)\cdot H_2fum$
Diflunisal	Fumaric acid	CH_3CN	Microcrystalline
Diflunisal	Pimelic acid	CH_3OH	$(Htmp^+)(dif^-)\cdot CH_3OH\cdot 0.5H_2O$
Diflunisal	Pimelic acid	CH_3CN	Multicrystalline
Diflunisal	Azeliac acid	CH_3OH	$(Htmp^+)(dif^-)\cdot CH_3OH\cdot 0.5H_2O$
Diflunisal	Azeliac acid	CH_3CN	Microcrystalline
Diflunisal	Sebacic acid	CH_3OH	$(Htmp^+)(dif^-)\cdot CH_3OH\cdot 0.5H_2O$
Diflunisal	Sebacic acid	CH_3CN	$(Htmp^+)_2(dif^-)_2\cdot H_2O$, crystals are poor diffractors, data not shown.
Diflunisal	Suberic acid	CH_3OH	Powder
Diflunisal	Suberic acid	CH_3CN	Non-diffracting needles
Diclofenac	Oxalic acid	CH_3OH	Crystallization of diclofenac
Diclofenac	Oxalic acid	CH_3CN	Powder
Diclofenac	Fumaric acid	CH_3OH	CURSAL
Diclofenac	Fumaric acid	CH_3CN	Powder
Diclofenac	Pimelic acid	CH_3OH	Powder
Diclofenac	Pimelic acid	CH_3CN	Thin fibers
Diclofenac	Azeliac acid	CH_3OH	Thin needles, non-diffracting
Diclofenac	Azeliac acid	CH_3CN	Multicrystalline
Diclofenac	Sebacic acid	CH_3OH	Crystallization of sebacic acid
Diclofenac	Sebacic acid	CH_3CN	Thin needles, non-diffracting
Diclofenac	Suberic acid	CH_3OH	Thin needles, non-diffracting
Diclofenac	Suberic acid	CH_3CN	Fibers

Table S2. Percentage contributions to the Hirshfeld surface areas of tmp/$Htmp^+$ in binary cocrystals/salts.

Cocrystal/salt	Crystal structure	O···H/H···O	N···H/H···N	C···H/H···C	H···H
$Htmp^+bz^-\cdot Hbz$ form I [a]	CUCSEY01	22.3	7.2	21.6	46.5
		19.7	7.8	21.6	47.5
$Htmp^+bz^-\cdot Hbz$ form II	CUCSEY10	20.9	6.7	14.7	51.1
$Htmp^+Hadi^-$	SEMNEE	23.2	7.7	7.5	54.0
$(Htmp^+)_2ter^{2-}\cdot H_2ter$	VADVOM	26.8	5.8	10.2	47.1
$Htmp^+Hglu^-$ [a]	CACBOY	25.2	7.5	8.5	50.9
$Htmp^+Hfum^-$ [a]	CURSAL	28.3	9.2	14.4	44.7
		26.5	9.0	17.1	44.7
		27.0	6.4	12.1	47.4
$Htmp^+Hketo^-\cdot 0.5H_2O$ [a]	KAXMIJ	30.8	7.0	12.3	45.3
$tmp\cdot H_2az$	KAXMOP	21.6	9.6	12.8	52.8
$Htmp^+Hmale^-\cdot CH_3CN$	this work	23.1	12.3	15.8	43.4
$Htmp^+Hmale^-\cdot$	QIKDIX	28.3	6.8	13.6	45.4
$(Htmp^+)(sub^{2-})_{0.5}(H_2sub)_{0.5}\cdot H_2O$	this work	24.6	3.8	8.2	60.2
$(Htmp^+)(adi^{2-})_{0.5}(H_2adi)_{0.5}\cdot 2H_2O$	this work	30.0	4.7	8.0	50.1
$Htmp^+H_2ptca^-\cdot CH_3CN$	this work	36.6	7.3	9.2	40.8
$(Htmp^+)_2(Hacn^{2-})\cdot 3H_2O$	this work	26.4	6.3	12.3	49.6
$Htmp^+Hmalo^-$	HAMYIE	27.8	5.1	8.6	50.9
$Htmp^+Hseb^-$	this work	27.8	7.9	10.3	53.1
$Htmp^+H_2tma^-\cdot 3H_2O$	KUMJIP	37.7	4.1	9.7	39.9
$Htmp^+Haz^-$	KAXNAC	28.5	7.9	9.2	53.3
$tmp\cdot H_2pim\cdot 0.5CH_3CN$ [a]	KAXNOC	20.5	12.6	11.3	52.7
		21.3	10.1	9.7	53.8

[a] two crystallographically independent $Htmp^+$

Table S3. Interaction energies for $Htmp^{+}Hglu^{-}$ (CACBOY) calculated with *Crystal Explorer*.

Interaction Energies (kJ/mol)
R is the distance between molecular centroids (mean atomic position) in Å.

Total energies, only reported for two benchmarked energy models, are the sum of the four energy components, scaled appropriately (see the scale factor table below)

	N	Symop	R	Electron Density	E_ele	E_pol	E_dis	E_rep	E_tot
	0	-	5.41	HF/3-21G	-213.4	-21.8	-27.4	9.8	-248.3
	1	-x, -y, -z	9.59	HF/3-21G	249.9	-31.5	-38.9	12.6	209.3
	1	-	10.01	HF/3-21G	-476.3	-120.9	-13.3	87.7	-504.8
	1	-	5.70	HF/3-21G	-210.7	-41.7	-21.1	8.2	-254.2
	1	-x, -y, -z	15.52	HF/3-21G	0.0	-14.0	0.0	0.0	-9.1
	1	x, y, z	8.80	HF/3-21G	182.0	-21.2	-29.6	11.0	153.9
	0	-x, -y, -z	6.91	HF/3-21G	118.2	-13.9	-12.7	1.0	100.7
	0	-	7.44	HF/3-21G	-121.8	-18.0	-17.0	12.4	-141.2
	0	-	7.09	HF/3-21G	-180.8	-16.9	-17.7	9.9	-203.1
	0	-x, -y, -z	11.11	HF/3-21G	100.4	-4.3	-2.1	0.1	97.7
	0	-x, -y, -z	9.70	HF/3-21G	87.0	-4.6	-21.7	10.7	74.8
	0	-x, -y, -z	5.98	HF/3-21G	46.4	-35.6	-71.6	35.9	-11.2
	0	-	11.17	HF/3-21G	-85.3	-6.0	-4.1	2.1	-92.8
	0	-x, -y, -z	13.37	HF/3-21G	0.0	-2.8	0.0	0.0	-1.8
	1	-	7.82	HF/3-21G	-302.3	-48.2	-14.4	24.9	-332.2
	1	-	7.57	HF/3-21G	-235.2	-27.4	-4.9	0.1	-261.9

Scale factors for benchmarked energy models
See Mackenzie et al. IUCrJ (2017)

Energy Model	k_ele	k_pol	k_disp	k_rep
CE-HF ... HF/3-21G electron densities	1.019	0.651	0.901	0.811
CE-B3LYP ... B3LYP/6-31G(d,p) electron densities	1.057	0.740	0.871	0.618

Table S4. Interaction energies for $Htmp^{+}Hadi^{-}$ (SEMNEE) calculated with *Crystal Explorer*.

Interaction Energies (kJ/mol)
R is the distance between molecular centroids (mean atomic position) in Å.

Total energies, only reported for two benchmarked energy models, are the sum of the four energy components, scaled appropriately (see the scale factor table below)

	N	Symop	R	Electron Density	E_ele	E_pol	E_dis	E_rep	E_tot
	0	-	10.38	HF/3-21G	-483.6	-124.9	-13.3	90.1	-512.9
	0	-	6.95	HF/3-21G	-214.5	-37.7	-9.1	1.2	-250.3
	1	x, y, z	8.17	HF/3-21G	186.9	-22.4	-39.4	21.0	157.4
	1	-x, -y, -z	6.32	HF/3-21G	66.4	-28.4	-62.7	26.7	14.3
	0	-x, -y, -z	12.43	HF/3-21G	0.0	-4.3	0.0	0.0	-2.8
	1	-	7.92	HF/3-21G	-110.2	-9.4	-17.9	7.6	-128.4
	1	-	5.48	HF/3-21G	-210.3	-17.0	-28.7	9.1	-243.7
	0	-	11.25	HF/3-21G	-87.6	-6.5	-4.0	3.0	-94.7
	1	-	6.39	HF/3-21G	-254.2	-41.7	-15.9	3.1	-297.9
	1	-x, -y, -z	9.54	HF/3-21G	87.9	-4.8	-23.6	12.1	75.0
	0	-x, -y, -z	10.15	HF/3-21G	254.1	-37.1	-38.4	15.2	212.5
	1	-	7.55	HF/3-21G	-313.3	-54.2	-17.0	35.7	-340.9
	1	-	7.06	HF/3-21G	-179.4	-18.2	-21.4	8.5	-207.1
	1	-x, -y, -z	7.68	HF/3-21G	107.1	-8.3	-5.1	0.0	99.1
	1	-x, -y, -z	10.66	HF/3-21G	99.5	-4.9	-4.3	1.3	95.4
	0	-x, -y, -z	15.67	HF/3-21G	0.0	-12.2	0.0	0.0	-7.9

Scale factors for benchmarked energy models
See Mackenzie et al. IUCrJ (2017)

Energy Model	k_ele	k_pol	k_disp	k_rep
CE-HF ... HF/3-21G electron densities	1.019	0.651	0.901	0.811
CE-B3LYP ... B3LYP/6-31G(d,p) electron densities	1.057	0.740	0.871	0.618

Table S5. Interaction energies for $Htmp^{+}H_2tma^{-}\cdot 3H_2O$ (KUMJIP) calculated with *Crystal Explorer*.

Interaction Energies (kJ/mol)
R is the distance between molecular centroids (mean atomic position) in Å.

Total energies, only reported for two benchmarked energy models, are the sum of the four energy components, scaled appropriately (see the scale factor table below)

	N	Symop	R	Electron Density	E_ele	E_pol	E_dis	E_rep	E_tot
	0	-	8.28	HF/3-21G	-468.1	-119.9	-14.4	106.5	-481.5
	0	-	8.52	HF/3-21G	4.3	-0.6	-1.1	0.1	3.1
	0	-x, -y, -z	11.72	HF/3-21G	61.4	-2.9	-11.7	2.7	52.4
	0	x, y, z	7.10	HF/3-21G	177.1	-14.0	-20.8	9.2	160.1
	0	-	5.88	HF/3-21G	-293.0	-60.6	-44.3	20.0	-361.6
	0	-	10.54	HF/3-21G	-257.4	-39.1	-20.6	132.1	-199.2
	0	-	5.51	HF/3-21G	-56.5	-16.0	-6.1	19.2	-57.9
	0	-x, -y, -z	8.89	HF/3-21G	246.2	-36.8	-20.9	2.9	210.4
	0	-x, -y, -z	7.25	HF/3-21G	79.0	-16.5	-32.0	12.9	51.4
	0	-	8.88	HF/3-21G	-158.0	-19.3	-15.6	47.6	-149.0
	0	-	8.82	HF/3-21G	-107.9	-4.9	-5.3	0.5	-117.5
	0	-	7.44	HF/3-21G	5.8	-1.4	-3.4	1.0	2.7
	0	-x, -y, -z	12.67	HF/3-21G	0.0	-1.8	0.0	0.0	-1.2
	0	-	5.17	HF/3-21G	-297.7	-44.8	-55.2	27.7	-359.7
	0	-x, -y, -z	11.06	HF/3-21G	102.3	-5.6	-6.3	1.4	96.1
	0	-	10.29	HF/3-21G	-117.2	-7.6	-5.2	5.2	-124.8
	0	-	6.80	HF/3-21G	-18.9	-4.1	-7.8	7.0	-23.3

Scale factors for benchmarked energy models
See Mackenzie et al. IUCrJ (2017)

Energy Model	k_ele	k_pol	k_disp	k_rep
CE-HF ... HF/3-21G electron densities	1.019	0.651	0.901	0.811

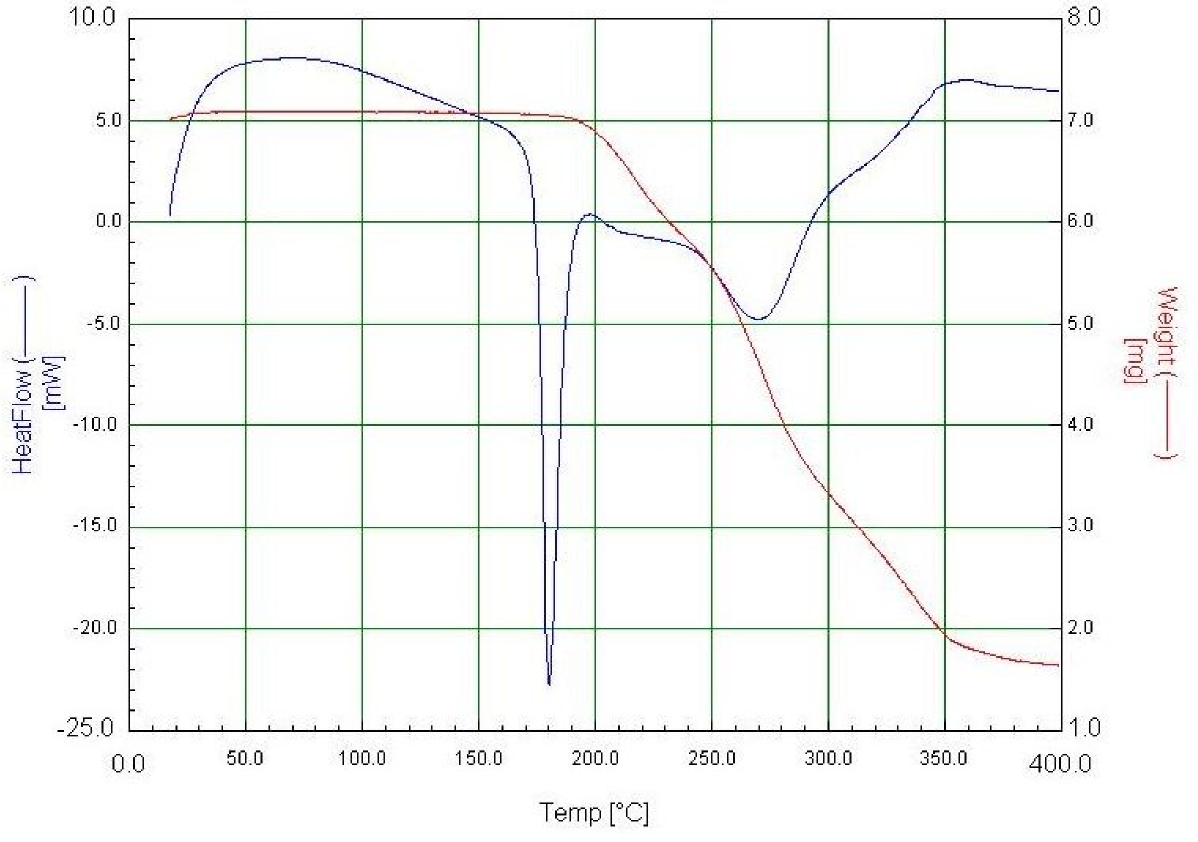

Figure S1. DSC plot of ($Htmp^+$)(dif^-)·H_2fum.

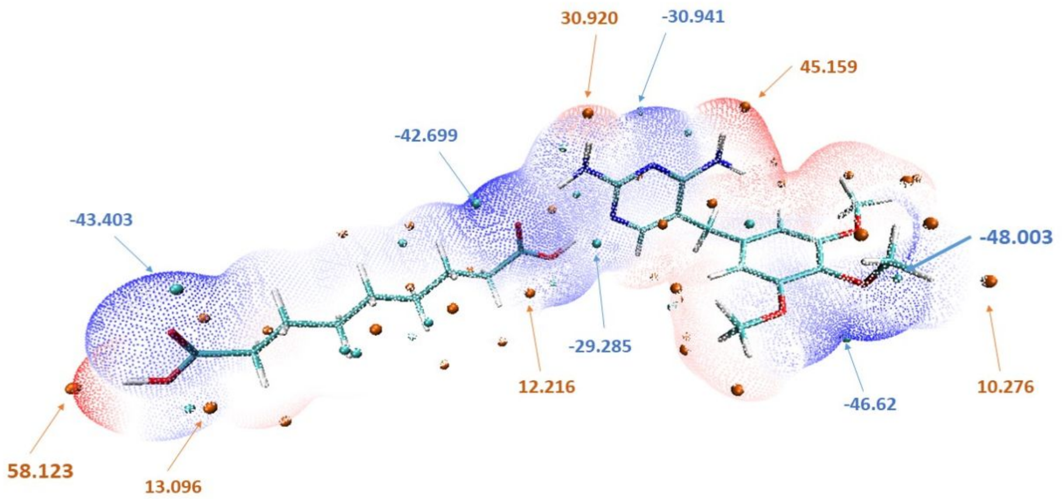

Figure S2. MEP of tmp·H_2az (CSD ref code KAXMOL) mapped onto the 0.001 a.u. electron density surface. Maxima and minima of the MEP are shown as red and cyan spheres.

Preparation of Binary Tmp Molecular Salts/Cocrystals with NSAIDs

$Htmp^+dif^-\cdot H_2O$ was crystallized by dissolving tmp (0.086 mmol) and Hdif (0.086 mmol) in the minimum amount of CH_3CN at 60 °C and slow evaporation at room temperature.

$Htmp^+dif^-\cdot CH_3OH\cdot 0.5H_2O$ crystallized from equimolar mixtures (0.086 mmol) of tmp, Hdif and pimelic acid, azeliac acid or sebacic acid in methanol.

$Htmp^+dic^-\cdot 1.5H_2O$ was prepared by dissolving equimolar amounts (0.086 mmol) of tmp and Hdic in the minimum amount of CH_3OH at 50 °C followed by slow evaporation at room temperature.

Preparation of Binary Tmp Salts/Cocrystals with Di- and Tricarboxylic Acids

$(Htmp^+)_2(Hacn^{2-})\cdot 3H_2O$ was prepared by dissolving equimolar amounts of tmp and H_3acn in the minimum amount of CH_3CN at 60 °C followed by slow evaporation at room temperature.

$Htmp^+Hmale^-\cdot CH_3CN$, **$(Htmp^+)(adi^{2-})_{0.5}(H_2adi)_{0.5}\cdot 2H_2O$**, **$Htmp^+H_2ptca^-\cdot CH_3CN$**, and **$(Htmp^+)(sub^{2-})_{0.5}(H_2sub)_{0.5}\cdot H_2O$** resulted from attempts to obtain ternary cocrystals of tmp, a carboxylic acid coformer and a sulfa drug. Equimolar amounts of the three coformers were dissolved in the minimum amount of CH_3CN at 60 °C and left to slowly to evaporate at room temperature. $Htmp^+Hmale^-\cdot CH_3CN$, $(Htmp^+)(adi^{2-})_{0.5}(H_2adi)_{0.5}\cdot 2H_2O$, and $tmpH^+H_2ptca^-\cdot CH_3CN$ crystallized from mixtures containing sulfamethizole, while $(Htmp^+)(sub^{2-})_{0.5}(H_2sub)_{0.5}\cdot H_2O$ formed in the presence of sulfioxazole.

$Htmp^+Hseb^-$ was obtained from an equimolar mixture of tmp, H_2seb and pyrazinamide in acetonitrile during attempts to obtain ternary cocrystals of tmp, a carboxylic acid coformer, and an anti-tuberculosis agent.

X-ray Structures of New Binary Tmp Molecular Salts/Cocrystals with NSAIDs and Carboxylic Acids

The hydrogen bonding motifs of $Htmp^+dif^-\cdot H_2O$, $Htmp^+dif^-\cdot 0.5H_2O\cdot CH_3OH$, $Htmp^+dic^-\cdot 1.5H_2O$, $Htmp^+Hseb^-$, $Htmp^+Hmale^-\cdot CH_3CN$, $(Htmp^+)(sub^{2-})_{0.5}(H_2sub)_{0.5}\cdot H_2O$, $(Htmp^+)(adi^{2-})_{0.5}(H_2adi)_{0.5}\cdot 2H_2O$, $(Htmp^+)_2Hacn^{2-}\cdot 3H_2O$, and $Htmp^+H_2ptca^-\cdot CH_3CN$ are shown in Figures S3 – S5. The deprotonated carboxyl groups of the coformers form the expected $R_2^2(8)$ synthon with the *N1H⁺,C2-NH₂* site of the $Htmp^+$ cation. Furthermore, the $R_2^2(8)$ homosynthon with a pair of C4-$NH_2\cdots$N3 hydrogen bonds is present in $(Htmp^+)(dif^-)\cdot H_2O$, $(Htmp^+)(dif^-)\cdot 0.5H_2O\cdot CH_3OH$, $(Htmp^+)(sub^{2-})_{0.5}(H_2sub)_{0.5}\cdot H_2O$, $(Htmp^+)(adi^{2-})_{0.5}(H_2adi)_{0.5}\cdot 2H_2O$, $Htmp^+Hmale^-\cdot CH_3CN$, and $Htmp^+H_2ptca^-\cdot CH_3CN$. In $Htmp^+dif^-\cdot H_2O$ hydrogen bonding between the $Htmp^+$ dimers and the water molecule of crystallization creates an additional $R_3^2(8)$ motif. The water molecule also interacts with the phenol group of the dif^- anion. The asymmetric unit of $Htmp^+dif^-\cdot 0.5H_2O\cdot CH_3OH$ contains two $Htmp^+dif^-$ entities (A and B), one water molecule and two methanol molecules. The water molecule of crystallization and the difluorophenyl ring of dif^- B are disordered over two positions with site occupancies of 0.8/0.2 and 0.75/0.25, respectively. The water molecule of crystallization stabilizes the C4-$NH_2\cdots$N3 homodimer between cation A and B by hydrogen bonding to C2-NH_2 of $Htmp^+$ A and C4-NH_2 of $Htmp^+$ B. One of the methanol molecules forms a hydrogen bond to the carboxylate group of dif^- A. The other methanol molecule interacts with two methoxy

substituents of $Htmp^+$ B and with the amino group at C2 of $Htmp^+$ A. Another hydrogen bond between the C4-amino group of $Htmp^+$ A and N3 of $Htmp^+$ B generates a motif of fused $R_1^2(5)$ and $R_4^3(10)$ rings.

The asymmetric unit of the salt of tmp and Hdic contains two $Htmp^+$ cations (denoted as A and B), two dic^- anions and three lattice water molecules. The two crystallographically independent cations are connected through a pair of C4-NH_2···N3/C2-NH_2···N3 hydrogen bonds. The second C4-NH_2 amino proton of A and a C4-NH_2 amino proton of B hydrogen bond with a water molecule of crystallization giving rise to $R_3^2(8)$ rings.

The ionic cocrystals $(Htmp^+)(sub^{2-})_{0.5}(H_2sub)_{0.5}·H_2O$ and $(Htmp^+)(adi^{2-})_{0.5}(H_2adi)_{0.5}·2H_2O$ have similar hydrogen bonding patterns with $Htmp^+$ cations being attached to both ends of the dianion. The neutral acid coformer forms a C-OH···$^-$OOC hydrogen bond with a dicarboxylate at both ends (Figure S4). Additional hydrogen bonding to one $[(Htmp^+)(sub^{2-})_{0.5}(H_2sub)_{0.5}·H_2O]$ or two $[(Htmp^+)(adi^{2-})_{0.5}(H_2adi)_{0.5}·2H_2O]$ water molecules of crystallization generates an $R_5^4(12)$ and $R_5^4(14)$ motif, respectively.

$Htmp^+Hmale^-·CH_3CN$ is a solvate of the known salt $Htmp^+Hmale^-$.[1] Both structures have the same hydrogen bonding pattern with the C4-NH_2···N3 homosynthon stabilized by additional C2-NH_2···O=(OH)C and C4-NH_2···O=(OH)C interactions and two $Hmale^-$ anions and two amino groups forming an $R_4^4(16)$ motif (Figure S5a).

The structure of $Htmp^+Hseb^-$ contains C4-NH_2···OCH_3, C2-NH_2···OCH_3, C4-NH_2···$^-$OOC and COOH···$^-$OOC interactions (Figure S5b).

Figures S5c and S5d show the structures of the salts of the two tricarboxylic acids, $(Htmp^+)_2Hacn^{2-}·3H_2O$ and $Htmp^+H_2ptca^-·CH_3CN$. In the former, two cations (A and B) interact with the deprotonated carboxyl group of the $Hacn^{2-}$ dianion. $Htmp^+$ A forms C2-NH_2···$^-$OOC and C4-NH_2···OH_2 hydrogen bonds. Hydrogen bonding between lattice water and the *C4-NH_2,N3* site of $Htmp^+$ B generates an $R_3^3(14)$ motif. In $Htmp^+H_2ptca^-·CH_3CN$, the C4-NH_2···N3 homosynthon is stabilized by additional hydrogen bonding between the carbonyl oxygen of one of the neutral carboxyl groups and the amino groups at C2 and C4 of $Htmp^+$. The second neutral carboxyl group interacts with the solvent molecule of crystallization.

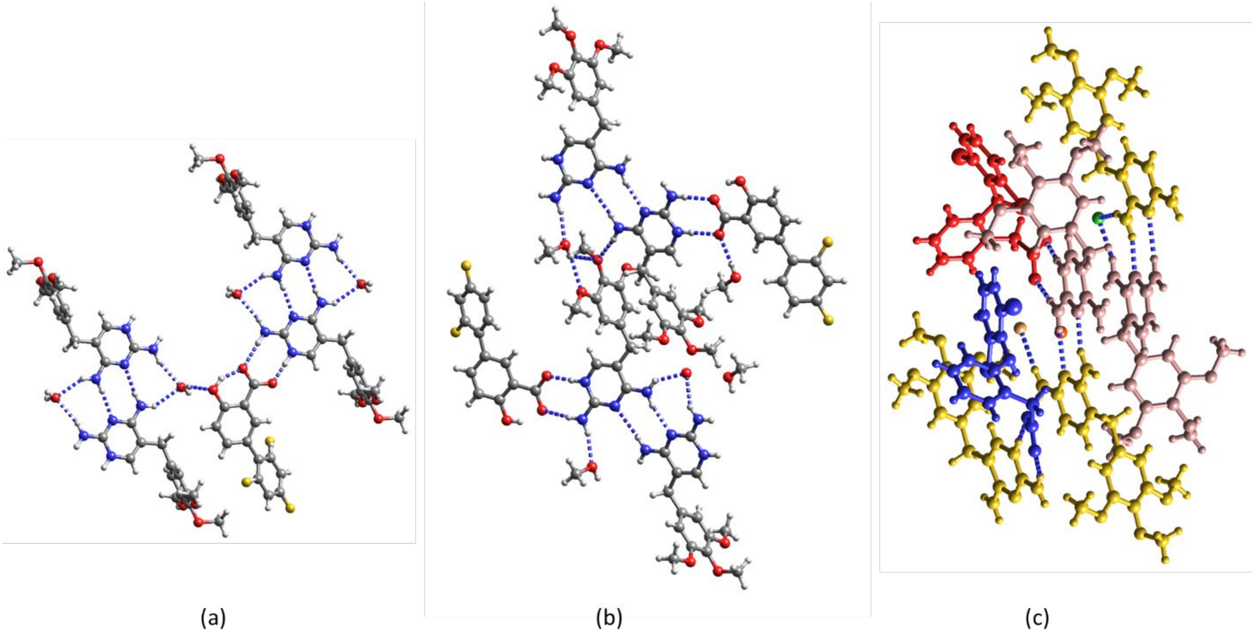

(a) (b) (c)

Figure S3. Hydrogen bonding motif in (a) $Htmp^+dif^-\cdot H_2O$, (b) $Htmp^+dif^-\cdot CH_3OH\cdot 0.5H_2O$ and (c) $Htmp^+dic^-\cdot 1.5H_2O$. For clarity, only the major components of the disordered water molecule and difluorophenyl ring of one of the dif^- anions is shown.

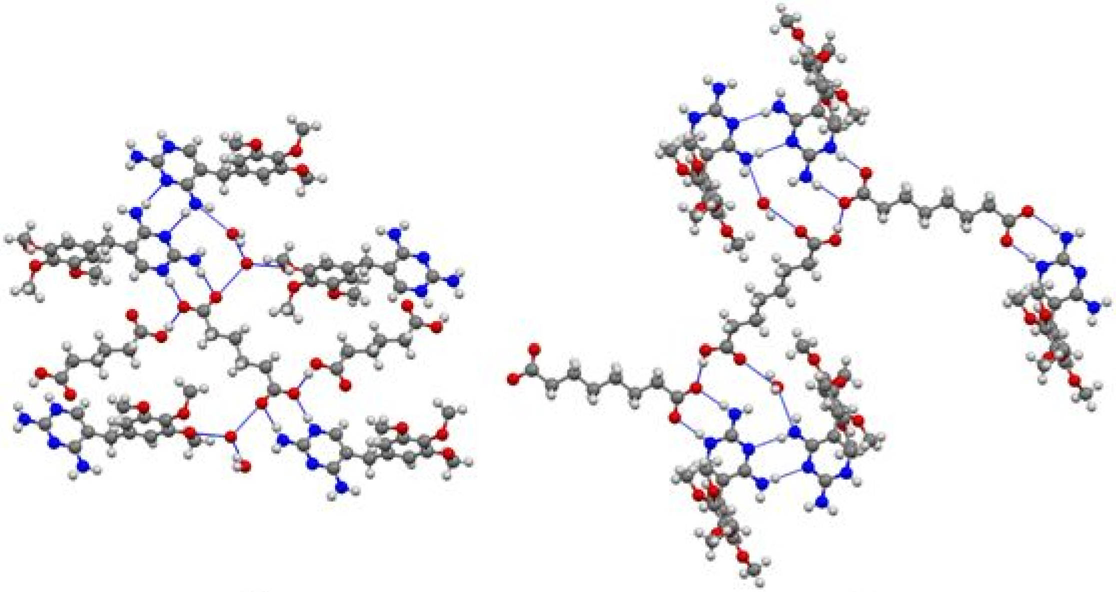

(a) (b)

Figure S4. Hydrogen bonding motif in (a) $(Htmp^+)(adi^{2-})_{0.5}(H_2adi)_{0.5}\cdot 2H_2O$ and (b) $(Htmp^+)(sub^{2-})_{0.5}(H_2sub)_{0.5}\cdot H_2O$. For clarity, only one component of the disordered water molecule is shown in (a).

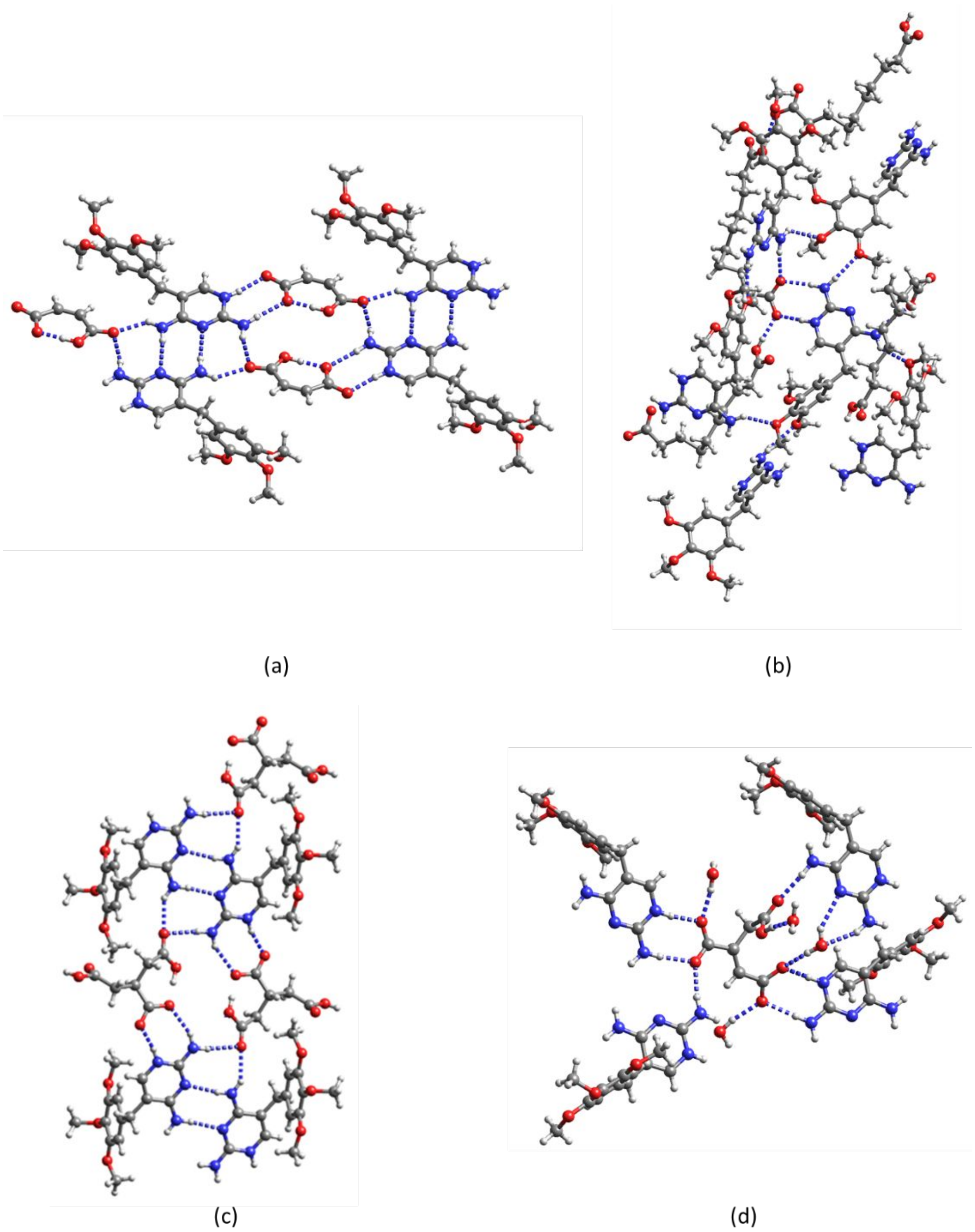

Figure S5. Hydrogen bonding motif in (a) $Htmp^{+}Hmale^{-}\cdot CH_3CN$, (b) $Htmp^{+}Hseb^{-}$, (c) $Htmp^{+}H_2ptca^{-}\cdot CH_3CN$, and (d) $(Htmp^{+})_2(Hacn^{2-})\cdot 3H_2O$.

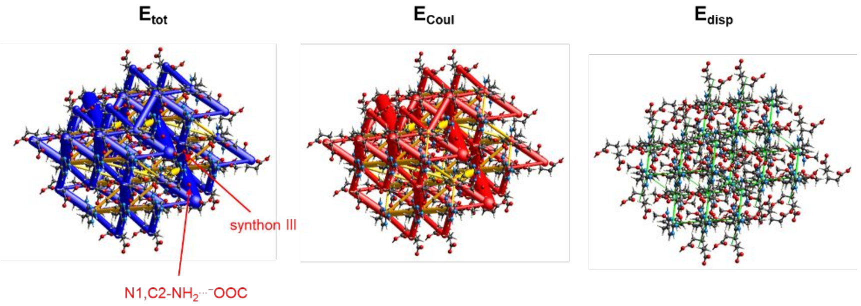

Figure S6. Energy framework diagrams for the total (E_{tot}), Coulomb (E_{Coul}), and dispersion (E_{disp}) energies for Htmp$^+$Hadi$^-$ (SEMNEE). The cylinder scale is 25 and the cut-offs are 50 for E_{tot} and E_{Coul} and 20 for E_{disp}. The energies were calculated at the HF/3-21G level of theory.

References

(1) Prabakaran, P.; Robert, J. J.; Thomas Muthiah, P.; Bocelli, G.;Righi, L. Amino-pyrimidine–carboxyl-(ate) interactions in trimethoprim maleate, an antifolate drug. *Acta Cryst*. **2001**, *C57*, 459–461.
